# Tailoring the clinical management of colorectal cancer by ^18^F-FDG PET/CT

**DOI:** 10.3389/fonc.2022.1062704

**Published:** 2022-12-22

**Authors:** Yang Shi, Meiqi Wang, Jiyu Zhang, Zheng Xiang, Can Li, Jingjing Zhang, Xing Ma

**Affiliations:** ^1^ Department of Gastroenterology, the First Affiliated Hospital of Zhengzhou University, Zhengzhou, China; ^2^ State Key Laboratory for the Prevention and Treatment of Esophageal Cancer, Zhengzhou University, Zhengzhou, China; ^3^ Academy of Medical Sciences, Zhengzhou University, Zhengzhou, China; ^4^ Department of Pathology, Henan Provincial People’s Hospital, Zhengzhou University People’s Hospital, Henan University People’s Hospital, Zhengzhou, China; ^5^ Department of Administration, The Affiliated Cancer Hospital of Zhengzhou University & Henan Cancer Hospital, Zhengzhou, China; ^6^ Department of Nuclear Medicine, the First Affiliated Hospital of Zhengzhou University, Zhengzhou, China; ^7^ Department of Nuclear Medicine, The Affiliated Cancer Hospital of Zhengzhou University & Henan Cancer Hospital, Zhengzhou, China

**Keywords:** FDG PET/CT, colorectal cancer, TNM staging, treatment monitoring, restaging

## Abstract

Colorectal cancer (CRC) is among the most commonly diagnosed gastrointestinal malignancies worldwide. It is inadequate to handle in terms of staging and restaging only based on morphological imaging modalities and serum surrogate markers. And the correct and timely staging of CRC is imperative to prognosis and management. When compared to established sequential, multimodal conventional diagnostic methods, the molecular and functional imaging ^18^F-FDG PET/CT shows superiorities for tailoring appropriate treatment maneuvers to each patient. This review aims to summarize the utilities of ^18^F-FDG PET/CT in CRC, focusing on primary staging, follow-up assessment of tumor responses and diagnostic of recurrence. In addition, we also summarize the technical considerations of PET/CT and the conventional imaging modalities in those patients who are either newly diagnosed with CRC or has already been treated from this cancer.

## 1 Introduction

Colorectal cancer (CRC) is the first most common and leading cause of gastrointestinal cancers (GI) worldwide, whose incidence and mortality rates accounted for 10% and 9.4%, respectively (1). Based on the statistics from International Researches Agency of Cancer (IRAC), the incidence and mortality of CRC would increase 40.1% and 43.4% by 2040 worldwide, respectively (2). Approximately 20% of CRC patients have initially diagnosed with metastases, mostly depositing in regional lymph nodes, liver, lung, and peritoneum (3). And the overall survival rate of patients is closely associated with stage at presentation; the 5-year survival rate drops drastically decreases from stage I (93%) to stage IV (8%) (4). Therefore, the precise staging of the CRC is essential for prognosis and effective therapy. The curative surgery has been remained as the “workhorse” at the early stage of cancers, and part of patients only with isolated liver metastasis (5). Nowadays, the standard of care regimens tends to be multidisciplinary, consisting of surgery, 5-fluorouracil-based chemotherapy, radiotherapy, targeted therapies, and PD-1/PD-L1 blockade biotherapy (5). Accurate pre- and postoperative staging are pivotal to tailor treatment avenues, which unequivocally improving the survival and quality of life. Variety of imaging techniques have been introduced for this purpose, with varying degree of success.

In the routine clinical practice, colonoscopy continues to be the preferred method for the diagnosis of colon cancer, because it allows tissue biopsies (6). In addition, a number of non-invasive imaging modalities, including as morphologic imaging such as computed tomography (CT), magnetic resonance imaging (MRI), and metabolic imaging such as positron emission tomography (PET), are presently used for staging colon cancer (7–9). Conventional imaging modalities, CT or MRI, are only based on the modifications of morphology of lesions, which could be invalid for cases mainly with metabolic changes. And serial elevated serum carcinoembryonic antigen (CEA) is usually coupled with false positives and false negatives (10). Recent studies showed that the combined-imaging modality is preferable for the detection and characterization of different malignant lesions including colorectum when compared with traditional imaging techniques (11). PET with flourine-18 fluorodeoxyglucose (^18^F-FDG PET/CT, hereafter refers as PET/CT) not only provides alternations of morphology, but also glycolytic metabolism changes based on glucose uptake, which increasing early diagnostic efficacy and visualize active tumor tissue (12). As for the molecular imaging, PET/CT represents a valuable ally for staging, therapeutic monitoring, and restaging for CRC patients (13, 14). However, the false positives were usually occurred when infection, inflammation, and other non-neoplastic conditions (15). Despite these drawbacks, PET/CT is irreplaceable in its ability to assess the abnormal metabolic activity precedes morphological change and to identify small sized malignant tumors in morphologically normal structures. This review attempts to discuss the role of PET/CT in the treatment of patients with CRC, which could pave the way for early accurate diagnosis and even individualized therapeutic strategies.

## 2 Evaluation of the preoperative TNM staging

**Figure 1:**
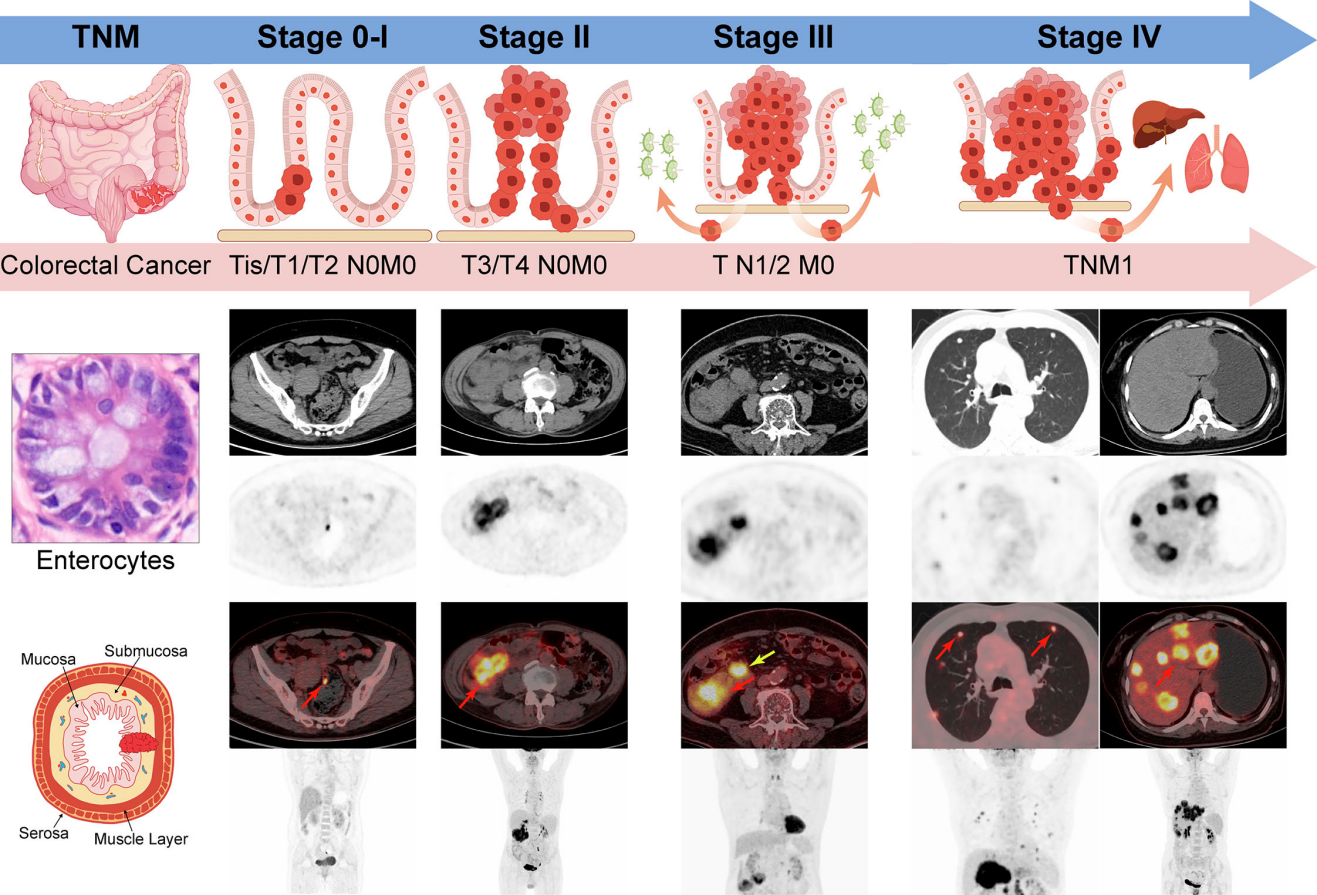
TNM stage of Colorectal cancer by AJCC 8th edition. Stage 0-I: Tumors spread within muscle layers without lymph nodes or distant deposits (Tis/T1/T2N0M0); Stage II: Tumors spread all layers or attached to nearby tissues without lymph nodes or distant deposits (T3/4N0M0); Stage III: Any T stage with the involvement of regional lymph nodes (TN1/2M0); Stage IV: Any T or N stage with distant metastasis (TNM1); Red arrow: the primary or metastatic tumors; yellow arrow: the metastatic lymph nodes.

Tumor-Node-Metastasis (TNM) staging system (AJCC) has provided the universal framework for optimal management of CRC patients since 1959 (16). The curative surgery is the mainstay of treatment based on accurate preoperative staging by the combination of T, N, and M indicators (**Figure 1**). Although the conventional imaging has served as the standard modality for this staging, PET/CT has been shown superior to evaluate the primary tumor, regional lymph nodes, and distant metastasis. Here we attempt to review and summarize the pros and cons of PET/CT in preoperative staging of CRC patients.

### 2.1 T-staging: The infiltration extent of primary tumor

Accurate preoperative evaluation of T-staging aids in selecting the related surgery approaches (17). Although limited spatial resolution of PET/CT confines its clinical application, there are still several strengths regarding preoperative assessment of T-staging. A meta-analysis including 2283 CRC patients from 28 studies shown the excellent performance for preoperative T-staging by PET/CT. The pooled specificity and AUC were 99% and 96%, respectively, which was significantly superior to CT (18). Other study demonstrated that the accurate preoperative T-staging was 94.3% except for only two tumors overestimation (19). As for the obstructive CRC, preoperative PET/CT colonography shows the particular advantages. Nagata K et al. demonstrated that PET/CT colonography could recognized all 13 primary CRC and 2 synchronous lesions proximal to the obstruction, while missed by other conventional imaging and optical colonoscopy more or less (20). Indeed, all lesions removed by single-stage procedure plays a vital role in the favorable outcomes of patients.

### 2.2 N-staging: Involvement of regional lymph nodes

Metastatic lymphadenopathy is indicated as a risk factor of recurrence and dismal survival in patients with CRC, which highlighting the merits of accurate detection and preoperative staging (21). The diagnosis of metastatic lymph nodes is generally based on a collection of morphological and density readout from the anatomic imaging techniques (CT & MRI). However, a plethora of positive lymph nodes tend to be not detected abnormality because of low density, small diameter, regular form and so on (22). Indeed, the overall accuracy of evaluation of N-staging by contrast enhanced CT (ceCT) has been reported merely from 59% to 71% (23, 24). Therefore, the alternative molecular imaging combined the morphological and functional techniques holds the promise for improving the diagnostic performance concerning metastatic deposits of CRC patients. Here we list several studies of the outstanding performance of PET/CT for this fields (**Table 1**) (22, 25–28).

**Table 1:** The comparison of diagnostic performance between CT and PET/CT for N-staging in CRC.

Ref.	Designs		Diagnostic Parameters (%)		
	Modality	No.	Sensitivity	Specificity	Accuracy
(22)	CT	220	58.7	64.8	62.3
	PET/CT		43.5	83.6	66.8
(23)	CT	473	87	29	59
	PET/CT		66	60	63
(25)	CT	370	38.4	95.5	65.0
	PET/CT		56.8	90.3	74.2
(26)	PET/CT	38	53.1	99.1	89.1
(27)	PET/CT	409	42.9	87.9	–

For detection of regional lymph node metastasis, a retrospective study including 370 CRC patients showed superiority of PET/CT in specificity compared with CT (83.6% vs. 64.8%, *p* = 0.000), while inferiority in sensitivity (43.5% vs. 58.7%, *p* = 0.029) (25). Consistently, Kwak et al. also reported higher specificity of PET/CT, while the comparable sensitivity compared to CT (28). Absence of functional information from CT has been recognized as the main drawback responsible for the lower specificity, while the slightly higher sensitivity could be due to the limited spatial resolution of PET/CT (29). Besides, there are still several studies indicated the possible reasons of PET/CT fail to detect the metastatic lymph nodes (1): the interference from physiological uptake by bowel and bladder (2); the proximity to the primary tumor so as to interfere the diagnosis of regional lymph node (14, 30).

### 2.3 M-staging: Distant metastases

Life expectancy of CRC patients declines to 71% when local regional lymph nodes involved, while plummet to 17% in the case of distant metastases (31). Therefore, the clinical significance of tumor staging is particularly regarding the assessment of distant metastases. Although PET/CT has been suggested utilized in few circumstances, including metastatic synchronous adenocarcinoma, and metachronous metastases with elevated serial CEA but negative colonoscopy or CT, various clinical trials pertaining to PET/CT have shown advantages for M-staging. Given that liver metastasis is the main site of advanced CRC patients, ineligibility for curative hepatic resection is based on extrahepatic deposits except for few resectable lung metastases. Ruers et al. demonstrated that PET/CT could reduce the futile laparotomies concerning liver metastasis from 45% to 28%, which decreasing unnecessary procedures and economic burden to a large extent (32). Other retrospective study based on Korean population reported that PET/CT was superior to CT in specificity (94% vs. 87%) and accuracy (93% vs. 86%), which was accounted for the specific metabolic changes by the former (33). Therefore, more clinical trials pertaining to PET/CT should be performed in order to pave the way for clinical utilization.

## 3 Assessment of the response to targeted therapies

The developments of targeted therapies were renewed and have flourished in the past two decades (34). Recent applications of such treatments in CRC have been exemplified by the approval of oral kinase inhibitors or monoclonal antibodies, *e.g.*, anti-angiogenesis (VEGF) and targeting epidermal growth factor receptor (EGFR). However, given only part of patients respond to these targeted therapies, it has been highly recommended to early stratify patients in order to reduce unnecessary toxicity and economic burden. Generally, the anatomic imaging modalities based on RECIST criteria, CT or MRI, have been utilized to monitor the response of cytoreductive or cytotoxic chemotherapy, while shows inappropriate for evaluating the cytostatic effect by the above-mentioned targeted therapy (35, 36). It is a common knowledge that the alternations of glucose metabolism within tumor precede the morphological changes by several weeks (37). Within this framework, we review the most widely accepted PET tracer and the only licensed biomarker, ^18^F-FDG (38), for assessing the responses to targeted therapies for patients with CRC.

**Figure 2:**
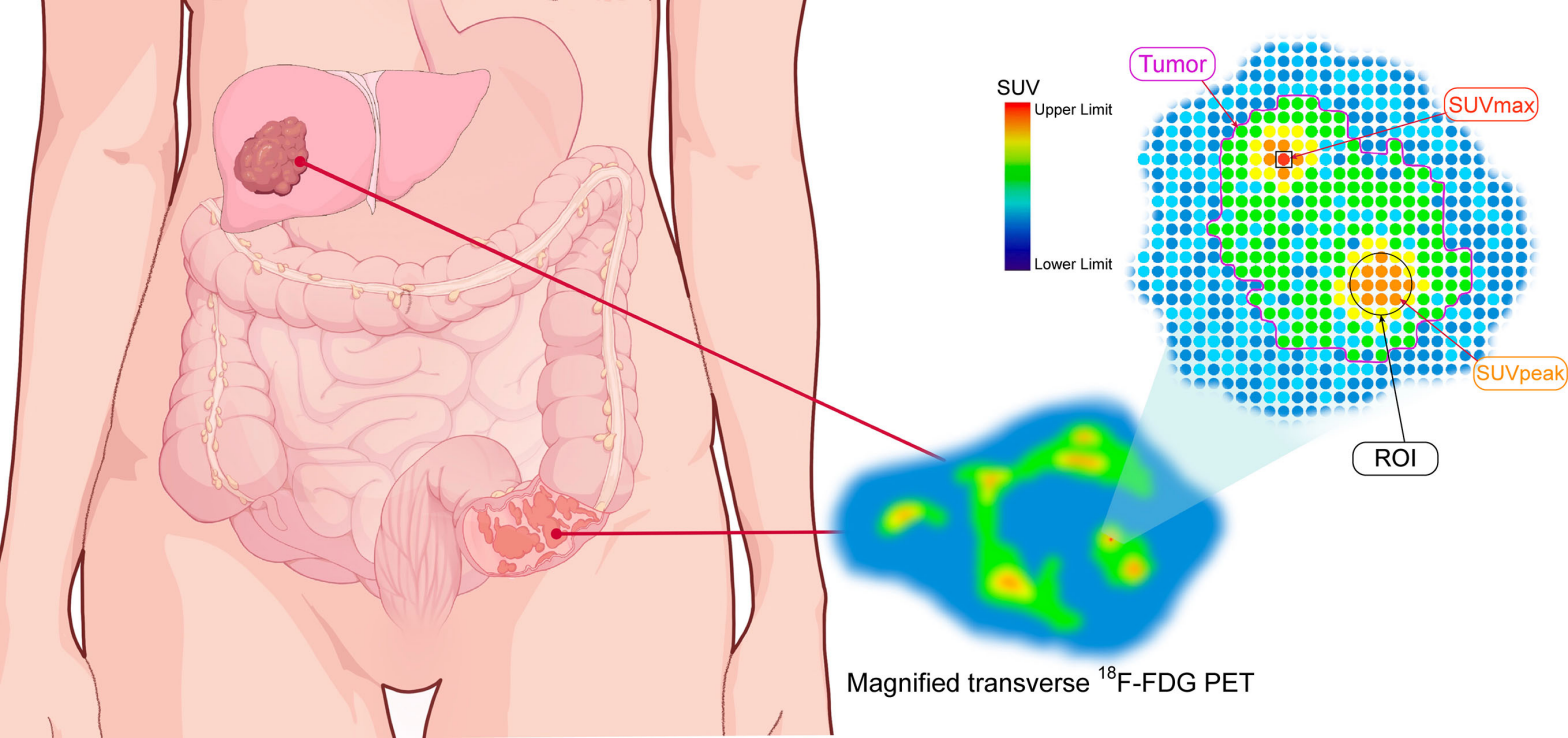
The schematic diagram of quantitative parameters of PET. The magnified transverse ^18^F-FDG PET image of radiotracer uptake in tumor (purple outline). SUVmax: maximum standardized uptake value (red spot within black square); ROI: region of interest (black circle); SUVpeak: the average SUV obtained from a 1mL sphere within the tumor.

Vascular endothelial growth factor (VEGF) targeted therapy disrupts tumor vasculature as well as inhibits angiogenesis, which have been exemplified by the first approved bevacizumab (Avastin) for metastatic colon cancer (mCRC) (39, 40). The semi-quantitative analysis of ^18^F-FDG uptake compared to normal liver was demonstrated to be instrumental for pathologic response prediction to bevacizumab in mCRC (41). Because of the quantitative trait of PET, quantitative measurement for early treatment response shows more attraction. Standardized uptake value (SUV) is the widely accepted quantitative metric for assessing treatment response in clinical PET/CT utility (**Figure 2**) (42). For example, the reduction of maximum SUV (SUVmax) more than 50% showed predictive value for early response to Bevacizumab in mCRC patients, with the larger the decline, the greater the efficacy (43). And the relative decrease of SUVmax more than 15% presented highly predictive for non-responders detection after one cycle of Sorafenib combined with capecitabine in mCRC patients (44).

Dysregulations of EGFR pathway have been extensively involved in carcinogenesis of CRC, such as metastasis, proliferation, and resistant to apoptosis (45–47). The cetuximab-based regimens targeting EGFR signaling cascade is clinically utilized as the third-line therapy for mCRC patients without KRAS mutation (48). The PET Response Criteria in Solid Tumors (PERECIST) criterion was proposed for assessing the early therapy response by the relative change of SULpeak, *i.e.*, peak SUV normalized to lean body mass (SUL) in a spherical 1 cm^3^ volume of interest (VOI) (49). Although treatment response using PERECIST predicted survival parameters (PFS & OS) at the end of cetuximab-based therapy (4 week) for mCRC, the innovative principle based on any eligible VOI more than 2 could ahead of this schedule after one week (50). And the early metabolic response based on the reduction of SUVmax more than 20%, fitted with EORTC criteria (51), could serve as the surrogate parameter for early clinical response under cetuximab in CRC patients (52). All cases reviewed here are summarized in **Table 2**.

**Table 2:** Evaluation of treatment response to targeted therapy by ^18^F-FDG PET/CT.

Ref.	Designs			Patient Info.				Criteria			Results & Objectives
	Drug	Type	Target	Type	Traits	No. Patients	No. Lesions	Guideline	Aim	Cutoff	
(42)	Bevacizumab + Chemo	anti-VEGF	VEGF	mCRC	LM	7	17	Δ^18^F-FDG uptake	CR	None uptake	Results: Response assessment = 70% Objectives: Pathologic outcome (% necrosis) prediction
(43)	Bevacizumab + Chemo	anti-VEGF	VEGF	mCRC	LM	11	–	–	Responders	ΔSUVmax >= -50%	Results: SUVmax from 8 (baseline) to 4 (after 1 cycle) Objectives: Early responders evaluation
(44)	Sorafenib + Chemo	VEGFR inhibitor	VEGF	mCRC	–	38	124	PERECIST adaptation	Responders	ΔSUVmax > -15%	Results: NPV of mR = 95%; PPV of mR = 72 % Objectives: Early non-responders detection
(50)	Cetuximab	anti-EGFR	EGFR	mCRC	*KRAS*-wt	27	85	–	Responders	(ΔSULpeak < 0 & SULpeak < 2)/VOI	Results: Favorable for PFS (*p*=0.001) & OS (*p*<0.001) Objectives: Early non-responders detection and survival prediction
(52)	Cetuximab	anti-EGFR	EGFR	mCRC	*KRAS*-wt	33	–	EORTC	Responders	ΔSUVmax > -20%	Results: Positive association: ΔSUVmax and ECR (OR=1.052, *p*=0.02) Objectives: Early non-responders detection

mCRC, metastatic colorectal cancer; LM, liver metastasis; Δ^18^F-FDG uptake, relative change of ^18^F-FDG uptake; CR, complete response; SUVmax, maximum standardized uptake value; ΔSUVmax = (SUVmax response - SUVmax baseline)/SUVmax baseline; NPV, negative predictive value; PPV, positive predictive value; mR, metabolic response; wt, wild type; SUL, SUV normalized to lean body mass; SULpeak, the average SUL within 1.2 cm diameter spheric VOI centered on the pixel with SULmax; ΔSULpeak, (SULpeak in S1 ‐ SULpeak in S0)/(SULpeak in S0), S1, baseline of study, S1, study at the end of the first week; VOI, volume of interest; OS, overall survival; PFS, progression-free survival; ECR, early clinical response; OR, odds ratio.

Therefore, detection of non-responders at early treatment stage could spare patients from the exposure of unnecessary toxicity and heavy economic burden.

## 4 Restaging of colorectal cancer

Most of CRC patients diagnosed at advanced staged would be suffered from recurrences, including liver, lung, and peritoneum metastases (21, 53). And serum markers and imaging modalities are utilized as the main examinations for those detection. Despite ceCT remains the clinical guideline recommendation, PET/CT is suggested in the workup of recurrent CRC patients with metachronous metastases, or elevated serial CEA but negative conventional imaging modalities and optical colonoscopy. Besides, PET/CT also presents the unique strengths for those kinds of scenarios.

Distinction of recurrent lesions from postoperative scar tissues is the important superiority of PET/CT. FDG uptake within recurrences could significantly reduce false positives and false negatives as well (54). The sensitivity and specificity of serum surrogate marker CEA has been demonstrated elevated when combined with PET/CT. One of the retrospective studies including 112 patients showed the early detection of recurrent CRC was 71% compared with 55% of CT when CEA level greater than 13 ng/mL (55). Actually, the level of CEA less than 5 ng/mL also presented excellent performance by PET/CT (56). Besides, the diagnostic accuracy of PET/CT for recurrent cases with elevated CEA was moderate (65-75%) in the case of negative findings by conventional imaging modalities (57). Moreover, with the aim of assess the impact of PET/CT in tailoring management in CRC patients with proven or suspected recurrence, and further to evaluate the impact of different management on the prognosis of patients. Scott et al. reported that PET/CT modified the therapeutic strategies of 65.6% CRC patients with symptomatic or residual lesions suggestive of recurrence. And the treatment maneuvers of 43.9% patients with resectable metastatic deposits in lung or liver have also been changed. Besides, the additional lesions of those patients showed progressive disease in 60.5% and 65.9% in the above-mentioned two groups of patients compared with conventional imaging techniques (36.2% & 39.2%), which indicated the value of PET/CT regarding the stratification of patients into curative or palliative avenues (58). In addition, there are also plenty of studies demonstrated that the outstanding performance for recurrent lesions by PET/CT in comparison with CT or MRI, which was generally accounted for the combination with functional imaging (59–61).

## 5 Conclusion and perspective

Effective and precise pre- and postoperative staging is essential for the best and individualized management of CRC patients, especially for those with metastatic lesions. Nowadays, the conventional imaging modalities are still the mainstay for staging processes, while insensitivity to the monitor the treatment response of targeted therapy. Given that the single trait of morphological imaging techniques, the additional function of metabolic evaluation by PET/CT shows particular advantages, including early detection for tumors, sensitivity to the treatment response to cytostatic drugs, and evaluation numerous body regions in a single process. This makes PET/CT particularly helpful in the staging of patients to exclude the presence of distant metastases as well as in the restaging. However, more research is required to standardize the ideal PET/CT timing in relation to the chosen therapy (immunotherapy or chemotherapy). And there is still a dearth of information regarding the diagnostic potency of PET/CT in the evaluation of CRC. Given that anatomic imaging techniques are superior to detect small lesions because of the high resolution, the combined molecular and morphological imaging PET/CT would broaden its clinical utility. In conclusion, PET/CT adds value to the diagnosis of metastatic lesions and may aid patients with CRC in selecting more appropriate treatment modalities.

## Author contributions

YS, XM, and JJZ formulated the design of this study; YS and XM wrote the manuscript; MW and CL revised the draft; JYZ drew the illustrations and figures; ZX assessed the histopathology of CRC. All authors contributed to the article and approved the submitted version.
